# The source of circulating selenoprotein S and its association with type 2 diabetes mellitus and atherosclerosis: a preliminary study

**DOI:** 10.1186/s12933-016-0388-3

**Published:** 2016-04-28

**Authors:** Shan-shan Yu, Li–li Men, Jia-ling Wu, Li-wei Huang, Qian Xing, Jun-jie Yao, Yong-bo Wang, Gui-rong Song, Hui-shu Guo, Guo-hua Sun, Yu-hong Zhang, Hua Li, Jian-ling Du

**Affiliations:** Department of Endocrinology, The First Affiliated Hospital of Dalian Medical University, Dalian, 116011 Liaoning China; Department of Diagnostic Ultrasound, The First Affiliated Hospital of Dalian Medical University, Dalian, 116011 Liaoning China; Department of Health Statistics, Dalian Medical University, Dalian, 116044 Liaoning China; Central Laboratory, The First Affiliated Hospital of Dalian Medical University, Dalian, 116011 Liaoning China; Department of Clinical Laboratory, The First Affiliated Hospital of Dalian Medical University, Dalian, 116011 Liaoning China; Department of Diagnostic Ultrasound, The Second Affiliated Hospital of Dalian Medical University, Dalian, 116023 Liaoning China; Department of Pharmacology, College of Pharmacy, Dalian Medical University, Dalian, 116044 Liaoning China

**Keywords:** Secreted selenoprotein S, Type 2 diabetes mellitus, Atherosclerosis

## Abstract

**Background:**

Selenoprotein S (SelS) is a transmembrane protein that is expressed in the liver, skeletal muscle, adipose tissue, pancreatic islets, kidney, and blood vessels. In addition to its transmembrane localization, SelS is also secreted from hepatoma HepG2 cells (but not L6 skeletal muscle cells, 3T3-L1 adipocytes, Min6 pancreatic β cells and human embryonic kidney 293 cells) and has been detected in the serum of some human subjects, with a detection rate of 31.1 %. These findings prove that serum SelS is secreted by hepatocytes. However, whether vascularly expressed SelS can be secreted has not been reported. Transmembrane SelS has been suggested to play different roles in the pathogenesis and progression of diabetes mellitus (DM) and atherosclerosis (AS), but the association of secreted SelS with DM and macroangiopathy remains unclear.

**Research design and methods:**

Supernatants were collected from human umbilical vein endothelial cells (HUVECs), human aortic vascular smooth muscle cells (HA/VSMCs) and human hepatoma HepG2 cells that were untransfected or transfected with the indicated plasmid and concentrated for western blotting. Serum samples were collected from 158 human subjects with or without type 2 DM (T2DM) and/or AS. Serum SelS levels were measured using an enzyme-linked immunosorbent assay.

**Results:**

Secreted SelS was only detected in the supernatants of hepatoma HepG2 cells. The SelS detection rate among the 158 human serum samples was 100 %, and the average SelS level was 64.81 ng/dl. The serum SelS level in the isolated DM subjects was lower than the level in the healthy control subjects (52.66 ± 20.53 vs 70.40 ± 21.38 ng/dl). The serum SelS levels in the DM complicated with SAS subjects (67.73 ± 21.41 ng/dl) and AS subjects (71.69 ± 27.00 ng/dl) were significantly increased compared with the serum SelS level in the isolated DM subjects. There was a positive interaction effect between T2DM and AS on the serum SelS level (P = 0.002). Spearman correlation analysis showed that the serum SelS level was negatively correlated with fasting plasma glucose.

**Conclusions:**

Vascular endothelial and vascular smooth muscle cells could not secrete SelS. Serum SelS was primarily secreted by hepatocytes. SelS was universally detected in human serum samples, and the serum SelS level was associated with T2DM and its macrovascular complications. Thus, regulating liver and serum SelS levels might become a new strategy for the prevention and treatment of DM and its macrovascular complications.

**Electronic supplementary material:**

The online version of this article (doi:10.1186/s12933-016-0388-3) contains supplementary material, which is available to authorized users.

## Background

Selenoprotein S (SelS, also known as Tanis, SEPS1 and VIMP) is a 21 kDa transmembrane protein with an extensive histological distribution. SelS is expressed in the liver, skeletal muscle, adipose tissue, pancreatic islets, kidney and blood vessels [1–6]. Studies have shown that SelS is closely associated with inflammation, oxidative stress and endoplasmic stress. It acts as a receptor for the acute phase inflammatory response protein serum amyloid A [2], and a NF-κB binding site is located within the SelS gene promoter region [4]. Moreover, SelS can regulate the production of inflammatory factors such as IL-1β and IL-6 [7]. SelS is a thioredoxin-dependent reductase that can reduce its substrate hydrogen peroxide (H_2_O_2_) or other peroxidases and combat the reactive oxygen species produced during the oxidative stress reaction [8]. Furthermore, SelS participates in the endoplasmic reticulum (ER)-associated protein degradation pathway by forming a complex with Derlin-1, ubiquitin ligase E3, p97 ATPase and selenoprotein K; this complex is responsible for the degradation of unfolded or misfolded proteins [9, 10].

The above biological characteristics of SelS suggest that it plays roles in the pathogenesis and development of diabetes mellitus (DM) and atherosclerosis (AS). Walder et al. [2] reported that hepatic SelS expression in the *Psammomys obesus*, a polygenic animal model of type 2 diabetes and metabolic syndrome, with impaired glucose tolerance and type 2 DM (T2DM) was lower than expression in *Psammomys obesus* with normal glucose tolerance. Hepatic SelS expression was inversely correlated with the circulating glucose and insulin levels. These authors found that SelS overexpression in hepatoma H4IIE cells reduced basal and insulin-stimulated glucose uptake, glycogen synthesis and glycogen content in vitro [11]. Furthermore, Walder et al. [2] showed that SelS expression in cultured C2C12 muscle cells and 3T3-L1 adipocytes was inhibited by glucose and insulin in a dose-dependent manner. However, Gao et al. [3] found that the overexpression of SelS in Min6 pancreatic β cells in pancreatic islets increased their resistance to H_2_O_2_-induced injury and increased their cell viability. In a clinical study, Karlsson et al. [12] analyzed SelS mRNA expression in the subcutaneous adipose tissues of T2DM patients and healthy individuals matched for age and body weight and found that the SelS mRNA in the subcutaneous adipose tissues of T2DM patients was significantly increased after hyperinsulinemic-euglycemic clamp experiments; in contrast, no significant change in expression was detected in the healthy control group. Subsequently, our group analyzed SelS mRNA expression in omental adipose tissues from T2DM patients and non-T2DM individuals and showed that SelS expression in these tissues was higher in T2DM patients than that in non-DM individuals and was positively correlated with the insulin resistance index [13]. The above studies indicated that membrane SelS was closely associated with the body glucose metabolic process. Briefly, SelS expression in the liver, adipose tissue, and skeletal muscle promoted the pathogenesis and development of DM and insulin resistance, whereas overexpression of SelS in pancreatic islets protected pancreatic islet β cells from oxidative stress-induced injury. Studies of SelS expression in blood vessels have also been recently reported. Our group showed that SelS overexpression protected human umbilical vein endothelial cells (HUVECs) from H_2_O_2_-induced injury [5]. Ye et al. [6] reported that the inhibition of SelS expression in primary vascular smooth muscle cells (VSMCs) increased H_2_O_2_- or tunicamycin-induced apoptosis. In conclusion, transmembrane SelS is closely associated with DM and AS and has advantageous and disadvantageous effects in different tissues and organs.

In addition to SelS transmembrane localization, Gao et al. [1] first detected secreted SelS in the culture media of hepatoma HepG2 cells and the serum of some human subjects, with a detection rate of 31.1 %. SelS secretion has not been detected in the supernatants of L6 skeletal muscle cells, 3T3-L1 adipocytes, Min6 pancreatic β-cells and human embryonic kidney 293 cells to date, indicating that serum SelS is secreted by hepatocytes. In addition to the expression of SelS in the liver, skeletal muscle, adipose tissue, pancreatic islets and kidney, SelS was recently shown to be expressed in the vascular endothelium and in vascular smooth muscle [5, 6]. However, whether SelS expression in the vascular endothelium and vascular smooth muscle is another source of secreted SelS is unknown. Transmembrane SelS is closely associated with DM and AS, but the association of secreted SelS with DM and macroangiopathy remains unclear. Therefore, this study analyzed SelS levels in the supernatants of vascular endothelial cells and vascular smooth muscle cells to investigate the source of serum SelS. Next, sera was collected from 158 human subjects to evaluate the serum SelS detection conditions. Finally, the SelS levels in patients with T2DM and AS were compared to investigate the association of secreted SelS with T2DM and AS.

## Methods

### Cellular experiments

#### Construction of the SelS recombinant plasmid

The human SelS gene (GenBank: NM_018445.5) fragment from the 104th to the 1205th nucleotide with a total length of 1102 bp was synthesized by Shanghai Sangon Biotech Co., Ltd., China. *Bam*HI and *Eco*RI restriction enzyme sites were added at the 5′ and 3′ ends of the fragment, respectively. Then, the SelS gene fragment and the pcDNA3.1(+) vector (5428 bp in length) were digested with *Eco*RI and *Bam*HI and ligated using T4 DNA ligase to construct the pcDNA3.1-SelS recombinant plasmid. After identification by restriction endonuclease analysis via agarose electrophoresis and sequencing, the pcDNA3.1-SelS recombinant plasmid was transiently transfected into cells using the Lipofectamine 2000 reagent (Invitrogen, USA).

### Cell culture and treatment

HUVECs, human aortic vascular smooth muscle cells (HA/VSMCs), and human hepatoma HepG2 cells were obtained from the American Type Culture Collection (ATCC, USA) and cultured in Dulbecco’s modified Eagle’s medium supplemented with 10 % fetal bovine serum, 100 U/ml of penicillin, and 100 μg/ml of streptomycin in a humidified 5 % CO_2_ atmosphere at 37 °C.

The above three cell types were inoculated into 10 cm dishes. Each cell type was divided into three groups as follows: normal control group (NC) without transfection, pcDNA3.1 vector control group (pc-VC) with pcDNA3.1 empty vector transfection, and pcDNA3.1-SelS group (pc-SelS) with pcDNA3.1-SelS recombinant plasmid transfection. The cells in the pc-VC and pc-SelS groups were transiently transfected with the corresponding plasmids for 24 h. The cells in the NC group were incubated for 24 h without transfection. Then, the cells were washed three times with serum-free Dulbecco’s modified Eagle’s medium to remove dead cells and subsequently cultured in 4 ml of serum-free Dulbecco’s modified Eagle’s medium for 24 h. The supernatants and cells in each group were carefully collected. The supernatants were centrifuged at 1000 rpm for 5 min and then transferred to ultrafiltration centrifuge tubes (Amicon^®^ Ultra-4 Centrifugal Filter Devices, Millipore, USA) with a cutoff molecular weight of 3 kDa. After concentration, the supernatants were washed with phosphate-buffered saline and concentrated again to 100 µl (concentration fold was 4 ml/100 µl = 40-fold) for western blotting to detect secreted SelS. The collected cells were lysed, and the total protein was prepared for western blotting to evaluate endogenous SelS expression in the three cell types and SelS expression after pcDNA3.1 vector and pcDNA3.1-SelS transfection. The protein concentration was determined using a BCA protein assay kit (KeyGEN, China).

### Western blotting analysis

A total of 30 μl of concentrated supernatant and cell lysates containing 30 µg of total protein were subjected to 12 % SDS–polyacrylamide gel electrophoresis and then transferred to polyvinylidene difluoride membranes (Millipore, USA). The polyvinylidene difluoride membranes were blocked in phosphate-buffered saline and Tween 20 containing 5 % non-fat dry milk powder for 1 h. Then, the membranes were incubated overnight at 4 °C with a rabbit anti-human SelS primary antibody (Abcam, USA) and subsequently incubated with a HRP-labeled goat anti-rabbit secondary antibody (Thermo Fisher Scientific, USA) at 37 °C for 1 h. The protein bands were detected using an enhanced chemiluminescence detection kit (Thermo Fisher Scientific, USA) and analyzed using the Alpha chemiluminescence gel imaging system FluorChem FC3 (ProteinSimple, USA).

### Human study

#### Subjects

The inclusion criteria for T2DM patients were as follows: patients aged between 40 and 70 years who met the World Health Organization criteria published in 1999 for the diagnosis and classification of DM.

The inclusion criteria for patients with AS and subclinical AS (SAS) were as follows: (1) AS—patients aged between 40 and 70 years who showed evidence of cardiovascular diseases (patients with histories of angina pectoris or myocardial infarction and a coronary CT angiography or coronary angiography showing coronary atherosclerotic plaques or stenosis), cerebrovascular diseases (patients with histories of cerebral thrombosis or hypertension-induced cerebral hemorrhage confirmed by CT or MRI), and/or peripheral artery diseases (patients with intermittent claudication and arterial ultrasonography of the bilateral lower limbs showing arteriosclerotic plaques or stenosis) and (2) SAS—patients aged 40–70 years with no previous histories or present characteristics of cardiovascular diseases, cerebral vascular diseases or peripheral artery diseases but with intima media thickness values of the conducting arteries (common carotid artery or femoral artery) ≥1.0 mm and/or atherosclerotic plaques detected by ultrasonography.

The exclusion criteria were as follows: (1) acute diabetic complications within the previous 6 months; (2) renal or hepatic dysfunction; (3) acute or chronic infections within the previous 6 months; and (4) tumors or other chronic diseases.

This study selected 82 patients with T2DM hospitalized in the Department of Endocrinology of the First Affiliated Hospital of Dalian Medical University between October 2013 and December 2014. The patients were divided according to the presence of AS and SAS into the diabetes complicated with AS group (DAS, n = 29), the diabetes complicated with SAS group (DSAS, n = 29) and the isolated DM group (IDM, n = 24). Patients hospitalized in the Department of Cardiology, Neurology, and Vascular Surgery of our hospital during the same period who met the above AS inclusion criteria but did not have T2DM were recruited into the isolated AS group (IAS, n = 27). Patients who met the SAS inclusion criteria but did not have T2DM were included in the isolated subclinical AS group (ISAS, n = 22) during physical examinations in our hospital during the same period. Additionally, 27 healthy subjects matched for age and gender with the above groups but without T2DM, AS, or SAS were selected to form the healthy control group (HC, n = 27) during physical examinations in our hospital during the same period. The study was approved by the Ethics Committee of the First Affiliated Hospital of Dalian Medical University.

### Clinical and laboratory examinations

Height, body weight and waist circumference (WC) measurements were obtained from all subjects and the corresponding body mass index (BMI) was calculated. Systolic blood pressure (SBP) and diastolic blood pressure (DBP) at the resting state were measured twice continuously, and the mean values were calculated. Fasting plasma glucose (FPG), glycosylated hemoglobin A_1C_ (HbA_1C_), total cholesterol (TC), triglyceride (TG), high density lipoprotein cholesterol (HDL-C), and low density lipoprotein cholesterol (LDL-C) were measured using routine laboratory techniques. Serum samples were collected and stored at −80 °C prior to use. Serum SelS levels were measured using an enzyme-linked immunosorbent assay kit (Antibodies Online, USA) according to the manufacturer’s instructions. All samples were analyzed in triplicate.

The intima media thickness of the common carotid artery and the femoral artery were measured using a GE Logiq9 ultrasound performed by designated physicians. The following locations were measured: (1) common carotid artery—the thickest location between 5 cm upstream and 5 cm downstream of the carotid bulb and (2) femoral artery—the thickest location within 10 cm upstream of the femoral artery bifurcation.

### Statistical analysis

SPSS 17.0 software was used for the statistical analysis. Normally distributed quantitative data are presented as the mean ± standard deviation, and analysis of variance (ANOVA) in a factorial design was used for the comparison of clinical data among groups. Qualitative data are presented as frequencies, and the χ^2^ test was used for comparisons of percentages among groups. A comparison of SelS levels among the six groups was performed using ANOVA; the LSD method was performed after ANOVA for comparisons of the SelS levels between any two of the six groups. The interaction effect between AS and T2DM on the serum SelS level was assessed using ANOVA in a factorial design. The correlations of the serum SelS concentration with clinical variables were examined via Spearman bivariate correlation analysis. P < 0.05 was considered statistically significant.

## Results

### Verification of the pcDNA3.1-SelS recombinant plasmid

**Fig. 1:**
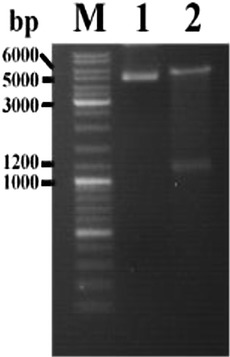
Restriction endonuclease analysis of the pcDNA3.1-SelS recombinant plasmid. M, 1, and 2 represent the DNA ladder mix, pcDNA3.1, and pcDNA3.1-SelS after *Eco*RI/*Bam*HI restriction enzyme digestion, respectively

The pcDNA3.1-SelS recombinant plasmid was verified by *Eco*RI/*Bam*HI restriction endonuclease digestion and agarose gel electrophoresis (Fig. 1). The SelS cDNA sequence (Additional file 1: Figure S1) in the pcDNA3.1-SelS recombinant plasmid was completely consistent with the sequence in GenBank (NM_018445.5). The results confirmed that the eukaryotic expression vector pcDNA3.1-SelS was constructed successfully.

### Detection of SelS expression in HUVECs, HA/VSMCs, HepG2 cell lysates and cell culture media

**Fig. 2:**
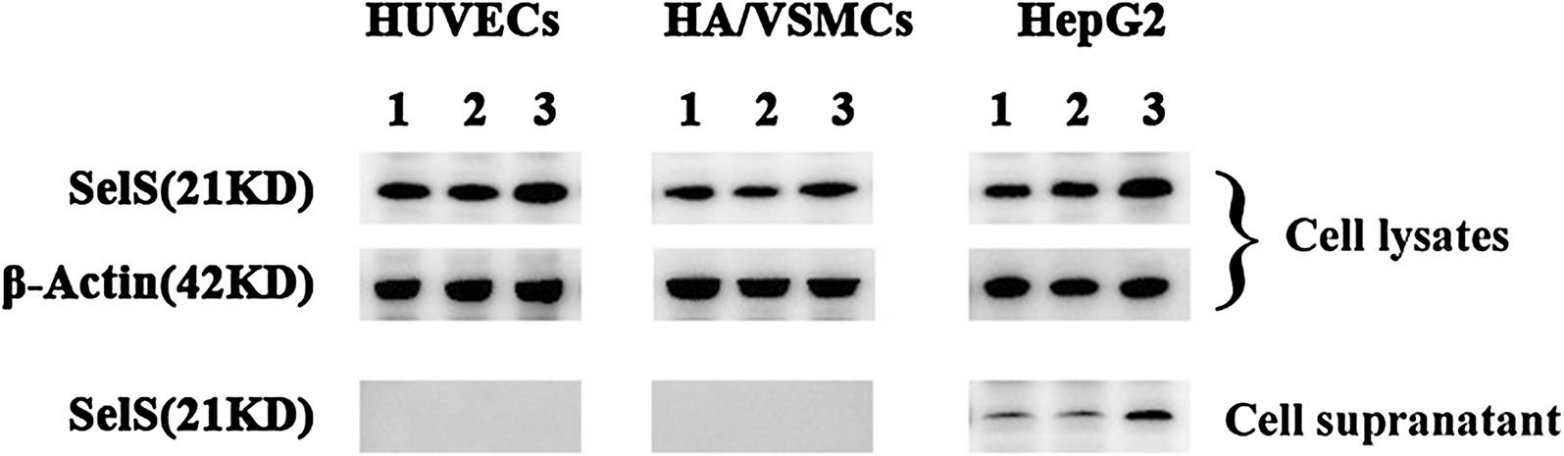
Detection of SelS in HUVEC, HA/VSMC, and HepG2 cell lysates and concentrated cell supernatants by western blotting. 1, 2, and 3 represent the normal control group *NC* without transfection, pcDNA3.1 vector control group *pc-VC* with pcDNA3.1 empty vector transfection, and pcDNA3.1-SelS group *pc-SelS* with pcDNA3.1-SelS recombinant plasmid transfection, respectively

The western blot analysis showed that SelS was naturally expressed in HUVECs, HA/VSMCs and HepG2 cells. SelS expression was unchanged after pcDNA3.1 empty vector transfection; however, its expression was increased after transfection of the pcDNA3.1-SelS recombinant plasmid into the above three cell lines. The concentrated cell culture media was analyzed using a western blot assay. Secreted SelS was only detected in the HepG2 cell supernatants. The secreted SelS level was unchanged after pcDNA3.1 empty vector transfection but was increased after transfection of the pcDNA3.1-SelS recombinant plasmid into HepG2 cells. In contrast, secreted SelS was not detected in the culture media of HUVECs or HA/VSMCs even after transfection with the pcDNA3.1-SelS recombinant plasmid (Fig. 2).

### Characteristics and comparisons of the clinical and laboratory variables among groups

WC, BMI, SBP, FPG, and TG were all higher in the T2DM group than in the non-T2DM group (subjects without T2DM) (P < 0.05). Age and SBP were higher in the SAS group than in the non-AS group (subjects without AS) (P < 0.05). Age, WC, BMI, and SBP were higher in the AS group than in the non-AS group (P < 0.05). WC, BMI, and TG were higher in the AS group than in the SAS group (P < 0.05) (Table 1).

**Table 1:** Clinical and laboratory characteristics of the study population

Group	Gender	Age^a, b, c^	WC^a, c, d, e^	BMI^a, c, d, e^	SBP^a, b, c, e^	DBP
	(Male/female)	(years)	(cm)	(kg/m^2^)	(mmHg)	(mmHg)
HC	27 (6/21)	56.22 ± 5.65	84.69 ± 9.98	24.16 ± 2.50	119.85 ± 13.56	77.44 ± 7.59
ISAS	22 (9/13)	56.73 ± 7.52	83.92 ± 10.44	24.64 ± 2.61	129.05 ± 12.67	80.14 ± 9.14
IAS	27 (13/14)	56.52 ± 6.72	87.13 ± 6.77	25.98 ± 2.67	129.07 ± 14.80	80.44 ± 6.91
IDM	24 (12/12)	49.71 ± 5.03	94.00 ± 9.03	26.91 ± 3.54	129.58 ± 17.32	81.25 ± 10.03
DSAS	29 (14/15)	58.17 ± 7.87	93.57 ± 10.01	26.14 ± 3.62	138.41 ± 17.39	81.00 ± 8.78
DAS	29 (14/15)	61.48 ± 7.88	98.28 ± 6.84	26.77 ± 2.93	141.10 ± 15.65	79.93 ± 8.56

Group	HbA1c	FPG^**e**^	Sqrt (TG)^a, d, e^	TC	LDL-C	HDL-C
	(%)	(mmol/L)	(mmol/L)	(mmol/L)	(mmol/L)	(mmol/L)
HC	–	5.37 ± 0.43	1.29 ± 0.40	5.45 ± 0.85	3.11 ± 0.63	1.29 ± 0.20
ISAS	–	5.23 ± 0.16	1.21 ± 0.24	5.02 ± 0.89	2.81 ± 0.48	1.21 ± 0.20
IAS	–	5.16 ± 0.40	1.23 ± 0.25	4.46 ± 1.15	2.54 ± 0.85	1.10 ± 0.27
IDM	8.42 ± 1.65	8.64 ± 2.85	1.38 ± 0.32	4.84 ± 1.20	2.72 ± 0.75	1.19 ± 0.30
DSAS	9.14 ± 1.89	7.90 ± 2.07	1.15 ± 0.25	4.51 ± 0.73	2.57 ± 0.56	1.12 ± 0.17
DAS	9.05 ± 1.42	9.32 ± 3.56	1.36 ± 0.46	5.07 ± 1.18	2.93 ± 0.82	1.23 ± 0.29

HC, ISAS, IAS, IDM, DSAS, and DAS represent the healthy control group, the isolated subclinical AS group, the isolated AS group, the isolated DM group, the diabetes complicated with SAS group and the diabetes complicated with AS group, respectively

^**a**^significantly different among non-AS, SAS, and AS at P < 0.05 based on ANOVA

^**b**^significantly different within groups between SAS and non-AS at P < 0.05 based on the LSD method after ANOVA

^**c**^significantly different within groups between AS and non-AS at P < 0.05 based on the LSD method after ANOVA

^**d**^significantly different within groups between SAS and AS at P < 0.05 based on the LSD method after ANOVA

^**e**^significantly different between subjects with and without T2DM

### Detection of serum SelS in all study subjects

Secreted SelS was detected in all serum samples from the 158 study subjects with a detection rate of 100 %. The box plot showed that the minimum SelS level was 12.75 ng/dl, the maximum level was 118.67 ng/dl, and the mean level was 64.81 ng/dl among all serum samples. The overall serum SelS level in the study population showed a norm al distribution (Additional file 2: Figure S2A–B).

### Comparisons of serum SelS levels among the six groups

**Fig. 3:**
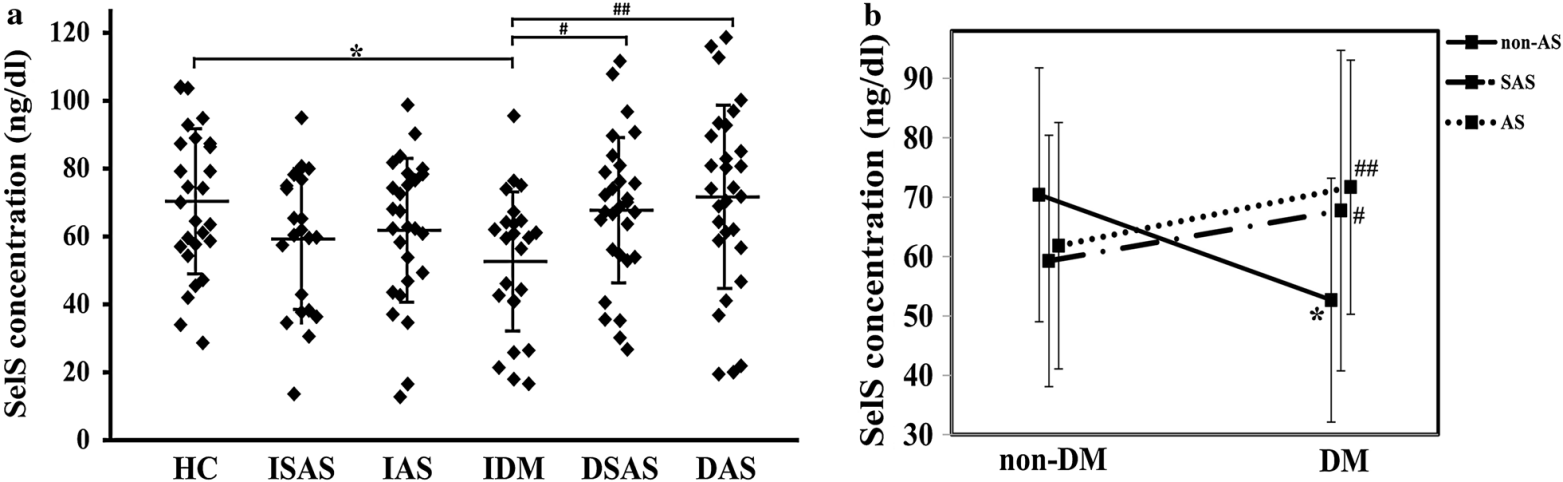
Serum SelS levels in different groups (**a**) and the interaction of AS and T2DM on the serum SelS concentration (**b**). HC, ISAS, IAS, IDM, DSAS, and DAS represent the healthy control group, the isolated subclinical AS group, the isolated AS group, the isolated DM group, the diabetes complicated with SAS group and the diabetes complicated with AS group, respectively. Values are expressed as the mean ± SD. *P < 0.01 compared to HC; ^#^P < 0.05 and ^##^P < 0.01 compared to IDM

The serum SelS level in the IDM group was lower than that in the HC group (52.66 ± 20.53 vs 70.40 ± 21.38 ng/dl, P = 0.005) (Fig. 3a). The serum SelS levels among the HC group, the ISAS group (59.26 ± 20.74 ng/dl) and the IAS group (61.83 ± 21.16 ng/dl) were not significantly different (P > 0.05) (Fig. 3a). The serum SelS levels were different in the three T2DM subgroups (P = 0.020) (Fig. 3a). The serum SelS levels in the DSAS group (67.73 ± 21.41 ng/dl, P = 0.015) and the DAS group (71.69 ± 27.00 ng/dl, P = 0.002) were significantly increased compared with the IDM group, but there was no significant difference between the DAS and DSAS groups (P > 0.05). The interaction plot of T2DM and AS on serum SelS (Fig. 3b) showed that the SelS levels did not differ among the non-DM groups, including the HC group, the ISAS group and the IAS group. Therefore, we speculated that the SelS concentrations in these three groups had the same starting point. The serum SelS level was decreased in patients with T2DM alone; however, when T2DM was complicated with SAS or AS, the serum SelS levels were significantly increased compared with the IDM group, suggesting that there was a positive interaction effect between T2DM and AS on the serum SelS level (P = 0.002).

### Spearman correlation analysis of the serum SelS concentration and clinical variables

A correlation analysis was performed on 51 study subjects in the HC and IDM groups. The serum SelS concentration was negatively correlated with WC (P = 0.020) and FPG (P = 0.028) but was not significantly correlated with the other clinical indicators (Table 2).

**Table 2:** Spearman correlation analysis between the serum SelS concentration and clinical variables

Clinical variables	r	P
Age (years)	0.204	0.151
WC (cm)	−0.324	0.020*
BMI (kg/m^2^)	−0.140	0.328
SBP (mmHg)	−0.158	0.269
DBP (mmHg)	−0.135	0.344
FPG (mmol/L)	−0.269	0.028*
TG (mmol/L)	−0.207	0.145
TC (mmol/L)	−0.071	0.621
LDL-C (mmol/L)	−0.050	0.729
HDL-C (mmol/L)	−0.115	0.421

* P < 0.05

## Discussion

SelS is a transmembrane protein that is localized to the cell membrane and ER membrane [4, 10]. In 2013, Bubenik et al. [14] reported a new subcellular localization for SelS, i.e., enriched at perinuclear speckles of hepatoma HepG2 cells. The authors co-stained HepG2 cells for SelS (green fluorescence) and a Golgi marker (golgin p97, red fluorescence) and showed a partial overlap between the two proteins (yellow fluorescence), suggesting that part of the perinuclear aggregated SelS was localized in the Golgi apparatus. In addition to its intracellular localization, SelS was also detected in the supernatant of HepG2 cells and human serum by Gao et al. [1]. The authors demonstrated that this secreted SelS was full length and was not proteolytically processed prior to secretion by HepG2 cells. Protein secretion by the majority of eukaryotic cells is achieved through the typical ER-Golgi secretory pathway [15]. Gao et al. [1] showed that SelS secretion was completely abolished after pretreatment of HepG2 cells with Brefeldin A (a well-known and specific ER-Golgi transport inhibitor). Combined with the endoplasmic reticulum and Golgi localization of SelS mentioned above [4, 14], these results indicated that the secretion of SelS by HepG2 cells was achieved through the endoplasmic reticulum-Golgi route. Gao et al. [1] demonstrated that SelS was secreted by hepatocytes but not L6 skeletal muscle cells, 3T3-L1 adipocytes, Min6 pancreatic β-cells and human embryonic kidney 293 cells. SelS was recently demonstrated to be expressed in the vascular endothelium and vascular smooth muscle in addition to the liver, skeletal muscle, adipose tissue, pancreatic islets and kidney [5, 6]. Because HUVECs and VSMCs were suggested to be capable of secreting a number of proteins associated with biological functions, such as cell proliferation, cell differentiation, cell adhesion, and cell signaling [16–19], we used these two cell types as model systems to investigate whether they could be another source of secreted SelS. This study showed that secreted SelS was not detected in the supernatants of naturally cultured HUVECs and HA/VSMCs, even after transfection with the pcDNA3.1-SelS recombinant plasmid. SelS was only detected in the supernatants of naturally cultured HepG2 cells, and the secreted SelS level increased after transfection with the pcDNA3.1-SelS recombinant plasmid. These results revealed that the vascular endothelium and vascular smooth muscle cells did not secrete SelS and that serum SelS was primarily secreted by hepatocytes.

Gao et al. [1] analyzed 209 human serum samples via ELISA. SelS was detected in 65 out of 209 human serum samples, for a detection rate of 31.3 %; the mean SelS level was 15.7 ng/mL [1]. In this study, we analyzed 158 human serum samples and detected SelS in all of them, for a detection rate of 100 %. The mean serum SelS level was 64.81 ng/dl. The overall serum SelS level in the study population showed a normal distribution. Differences in the race and region of the human subjects used in Gao’s study and ours might have contributed to the inconsistent results. The human subjects in Gao’s study were Caucasian (the white race) while in our study were Mongoloid (the yellow race). Additionally, the primary SelS antibody and SelS standard solution used in ELISA assay was created by Gao et al. [1] themselves while the ELISA assay kits used in our study were commercially available and relatively mature in technology, which might be another reason for the inconsistent results.

Hepatic SelS was closely associated with body glucose metabolism. Hepatic SelS expression in *Psammomys obesus* with impaired glucose tolerance and T2DM was lower than that in *Psammomys obesus* with normal glucose tolerance [2], and HepG2 SelS expression was suppressed by glucose in a dose-dependent manner in vitro [3]. The association between serum SelS and glucose metabolism was first analyzed by Gao et al. [1]. These authors detected and compared serum SelS levels in healthy individuals (41.7 %, 27.8 ng/ml), type 1 DM patients (23.2 %, 34.0 ng/ml), and T2DM patients (27.9 %, 34.3 ng/ml) and found that the average SelS level among these three groups did not differ significantly. In this study, we demonstrated that the serum SelS level in isolated T2DM patients was lower than that in healthy subjects and was negatively correlated with the fasting plasma glucose concentration. This finding was consistent with the aforementioned changes in hepatic SelS expression and HepG2 cells, indicating that serum SelS was secreted by hepatocytes. Gao et al. [11] found that SelS overexpression in hepatoma H4IIE cells reduced basal and insulin-stimulated hepatic glucose utilization and that this effect was even more evident under insulin stimulation. Additionally, the authors showed that SelS overexpression antagonized the inhibitory effect of insulin on phosphoenolpyruvate carboxykinase, which is a rate-limiting enzyme in the hepatic gluconeogenesis process, thereby increasing the hepatic glucose output. As a result, the reduction in the serum SelS level in T2DM patients in our study might be a body defense response to relieve the high glucose status and increase insulin sensitivity. These results suggest that reducing the hepatic and serum SelS levels might become a strategy for the prevention and treatment of T2DM.

SelS belongs to the selenoprotein family, which consists of glutathione peroxidases or thioredoxin reductases that contain one selenocysteine [20, 21]. Glutathione peroxidase activity was markedly augmented in rodent models with both diabetes and myocardial infarction compared to diabetes or myocardial infarction alone [22]. Moreover, pro-angiogenic cells were reported to exhibit lower levels of cytoprotective genes, including glutathione peroxidase [23]. These results indicate that SelS may be involved in the pathogenesis and development of diabetic macroangiopathy. However, an association between hepatic SelS and macroangiopathy has not been reported to date. In this study, we analyzed the association between serum SelS with AS and DM complicated with AS and showed that the serum SelS levels in the T2DM patients with SAS or AS did not differ from the level in healthy individuals. Intriguingly, among the T2DM groups, the serum SelS levels in the patients with DSAS or DAS were significantly higher than the level in T2DM patients. Additionally, there was a positive interaction effect between T2DM and AS on the serum SelS level. These results indicate that serum SelS might be involved in the pathogenesis and development of AS in T2DM patients and indirectly suggest that hepatic SelS might be associated with AS. However, the association between hepatic SelS and diabetic macroangiopathy requires further investigation. Recently, Cox et al. [24] analyzed the association between 10 single nucleotide polymorphisms (SNPs) in the SelS gene with the risk for AS and subclinical AS by examining 1220 European-Americans from the Diabetes Heart Study. The authors found that the SelS SNPs rs28665122, rs4965814, rs28628459, rs7178239, and rs12917258 were associated with subclinical AS, whereas the SelS SNPs rs4965814, rs28628459, and rs9806366 were associated with AS. The studies conducted by Cox et al. [24] and our group suggest a potential role for SelS gene polymorphisms in predicting the occurrence of macrovascular complications in T2DM patients. Thus, reducing liver and serum SelS levels may become new interventions for the prevention and treatment of diabetic macroangiopathy.

Serum amyloid A can interact with SelS; this interaction has been confirmed by a yeast 2-hybrid screen and surface plasmon resonance analysis [2]. Additionally, serum amyloid A is a risk factor for the occurrence of macroangiopathy in T2DM patients [25]. Therefore, we speculated that serum amyloid A might be related to the involvement of SelS in the pathogenesis of diabetic macroangiopathy. Furthermore, a NF-κB binding site is located within the SelS gene promoter [4]. Thus, as a target of NF-κB, SelS might participate in the occurrence and development of diabetic macroangiopathy through the NF-κB signaling pathway. However, the function of hepatic and serum SelS in DM and AS as well as the underlying mechanisms requires validation in animal models and warrants further investigation.

### Study limitations

There are several limitations in our study. First, we and Gao et al. [1] performed in vitro detections of whether the cells of corresponding tissues could secrete SelS according to the histological distribution of SelS. The results showed that SelS was not secreted by adipocytes, skeletal muscle cells, human embryonic kidney cells, pancreatic islet β cells, vascular endothelial cells, and vascular smooth muscle cells but was secreted by hepatocytes. Thus, we concluded that serum SelS was secreted from the liver. However, whether serum SelS is solely secreted from the liver remains unclear, and further studies using animal models with selective knockout of the hepatic SelS gene are required. Next, this study was retrospective; therefore, we could only investigate whether serum SelS was associated with T2DM and its macrovascular complications rather than draw a conclusion regarding the causal relationship between these two factors. Finally, the sample size in the present study was limited, and this study could be regarded as a preliminary study. Therefore, our results require further validation in a prospective randomized controlled trial with a larger sample size.

## Conclusions

Vascular endothelial cells and vascular smooth muscle cells did not secrete SelS, and serum SelS was primarily secreted by hepatocytes. SelS was universally detected in human serum samples, and the serum SelS level was associated with T2DM and its macrovascular complications. The reduction of liver and serum SelS levels may become a new target for the prevention and treatment of T2DM and its macrovascular complications.

## Additional files

10.1186/s12933-016-0388-3 Sequencing analysis of the pcDNA3.1-SelS recombinant plasmid.
10.1186/s12933-016-0388-3 Box plot (**A**) demonstrating the detection of the serum SelS concentration in the study population via an enzyme-linked immunosorbent assay (ELISA) and a histogram (**B**) of the serum SelS distribution in all subjects.

## Abbreviations

SelS: selenoprotein S
H_2_O_2_: hydrogen peroxide
ER: endoplasmic reticulum
DM: diabetes mellitus
AS: atherosclerosis
T2DM: type 2 DM
HUVECs: human umbilical vein endothelial cells
VSMCs: vascular smooth muscle cells
SAS: subclinical AS
DAS: diabetes complicated with AS
DSAS: diabetes complicated with SAS
IDM: isolated DM
IAS: isolated AS
ISAS: isolated subclinical AS
HC: healthy control
WC: waist circumference
BMI: body mass index
SBP: systolic blood pressure
DBP: diastolic blood pressure
FPG: fasting plasma glucose
HbA1C: glycosylated hemoglobin A1C
TC: total cholesterol
TG: triglyceride
HDL-C: high density lipoprotein cholesterol
LDL-C: low density lipoprotein cholesterol
NC: normal control
pc-VC: pcDNA3.1 vector control
pc-SelS: pcDNA3.1-SelS
ANOVA: analysis of variance
ELISA: enzyme-linked immunosorbent assay
SNPs: single nucleotide polymorphisms
