# Supplementary figures and images for: The source of circulating selenoprotein S and its association with type 2 diabetes mellitus and atherosclerosis: a preliminary study
Source: Cardiovasc Diabetol. 2016 Apr 28;15:70. doi: 10.1186/s12933-016-0388-3 (PMC4849094; doi:10.1186/s12933-016-0388-3)

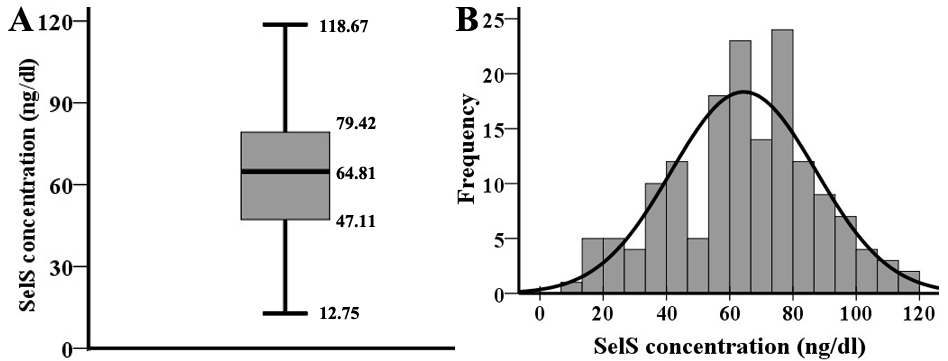

Supplement: Supplementary file 1 — 10.1186/s12933-016-0388-3 Sequencing analysis of the pcDNA3.1-SelS recombinant plasmid. [file 12933_2016_388_MOESM1_ESM.tif]

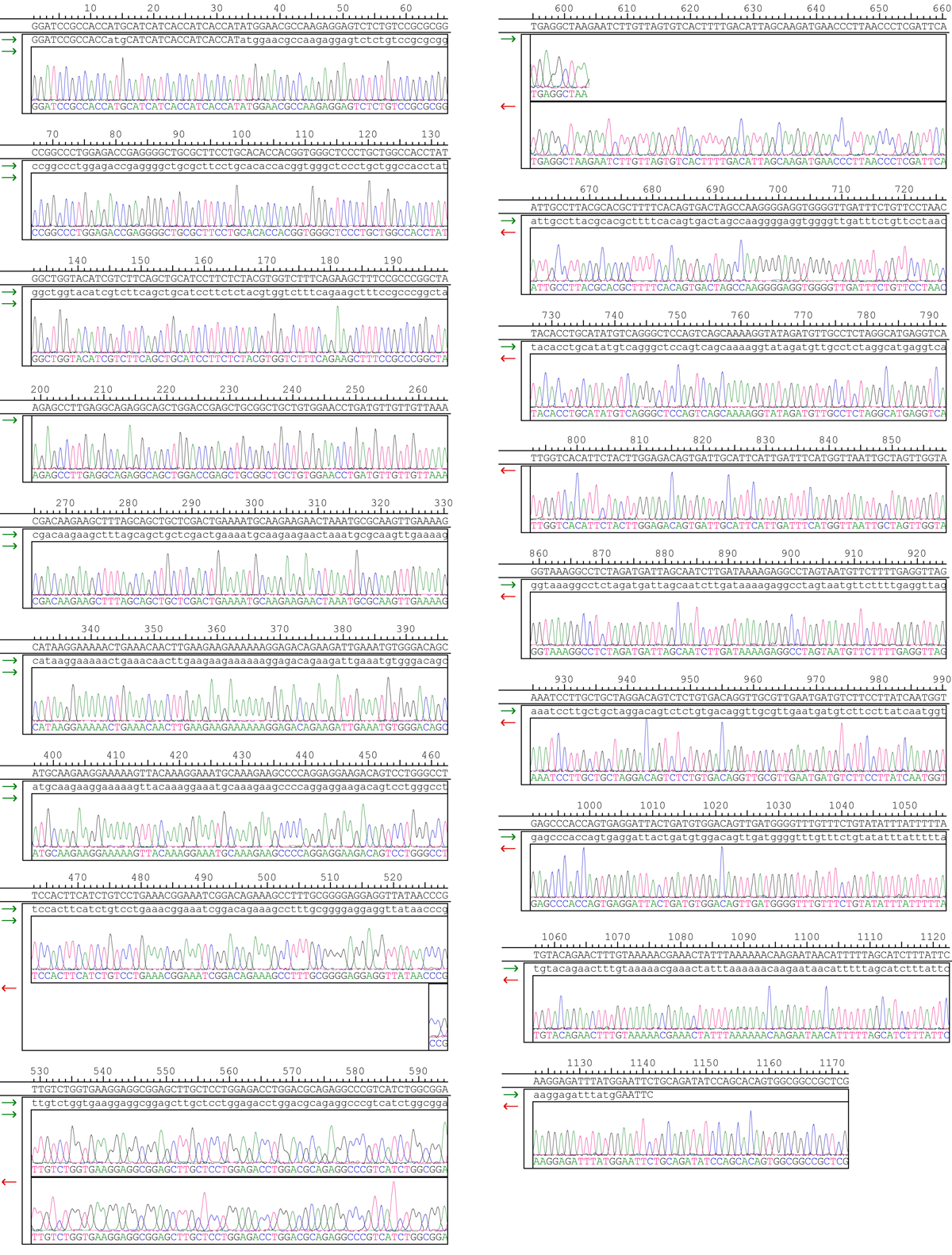

Supplement: Supplementary file 2 — 10.1186/s12933-016-0388-3 Box plot (A) demonstrating the detection of the serum SelS concentration in the study population via an enzyme-linked immunosorbent assay (ELISA) and a histogram (B) of the serum SelS distribution in all subjects. [file 12933_2016_388_MOESM2_ESM.tif]
